# Systematic establishment of approaches to the detection of equine macrophage polarization and their application in pathogenic infection

**DOI:** 10.1128/spectrum.02916-25

**Published:** 2026-07-06

**Authors:** Kewei Chen, Cheng Du, Yingyi Duan, Kui Guo, Diqiu Liu, Fengxue Wang, Xiaojun Wang, Yongjun Wen

**Affiliations:** 1 Key Laboratory for Clinical Diagnosis and Treatment of Animal Diseases of Ministry of Agriculture, College of Veterinary Medicine, Inner Mongolia Agricultural University, Hohhot, China; 2 State Key Laboratory of Animal Disease Control and Prevention, Harbin Veterinary Research Institute, Chinese Academy of Agricultural Sciences 111613, Harbin, China; Wannan Medical University, Wuhu, Anhui, China

**Keywords:** equine macrophages, macrophage polarization, infection, CD80, CD163, EIAV, EAV, EHV

## Abstract

Macrophage phenotypic adaptation critically regulates inflammatory balance during infection; however, progress in equine immunology has been limited by a lack of specific tools for standardized identification. To address this gap, we established a reliable, antibody-based detection method for characterizing equine macrophage M1-like and M2-like marker profiles. After initially validating canonical marker genes via qPCR, we developed specific monoclonal antibodies targeting the differentially expressed surface proteins CD80 (M1-like) and CD163 (M2-like). These novel antibodies enabled the creation of a multi-modal detection approach combining flow cytometry, western blotting, and qPCR. Applying this methodology *in vitro* revealed distinct pathogen-specific marker profiles: *Salmonella abortus equi*, equine herpesvirus (EHV-1), and equine arteritis virus (EAV) promoted an early M1-like profile, whereas an attenuated equine infectious anemia virus (EIAV) strain drove an M2-like phenotype. Ultimately, this work provides a validated detection system for equine macrophage phenotyping, establishing a critical foundation for future research into host-pathogen interactions and targeted therapeutics.

**IMPORTANCE:**

Macrophage phenotypic adaptation plays a critical role in infectious diseases, as pathogens often manipulate these states to evade immune responses or drive damaging inflammation. Accurately monitoring these functional shifts is vital for guiding disease treatment and evaluating vaccines. However, standardized detection tools for equine macrophages have been lacking. In this study, we established a reliable, antibody-based method utilizing novel monoclonal antibodies against equine CD80 and CD163 to identify M1-like and M2-like marker profiles. This straightforward and highly specific approach overcomes the limitations of previous indirect methods, providing a critical, accessible tool for assessing macrophage responses to equine pathogens and advancing veterinary immunology.

## INTRODUCTION

The action of monocytes and macrophages has been reported to be an important weapon of the host innate immune response against various pathogens. The action of these cells has been extensively studied in the context of infectious diseases caused by viruses, protozoa, bacteria, and fungi (1–3). Macrophages exhibit diverse functions determined by their polarization states. Upon activation by pathogens or injury, they can polarize into classically activated (M1) or alternatively activated (M2) phenotypes (4–6). M1 macrophages, induced by IFN-γ and LPS, are pro-inflammatory and antimicrobial, producing cytokines such as IL-1β, IL-12, and TNF-α (7, 8). In contrast, M2 macrophages, stimulated by IL-4, IL-10, or IL-13, are anti-inflammatory and promote tissue repair, primarily secreting IL-10 and TGF-β (9). M2 macrophages are further divided into subtypes (M2a, M2b, and M2c) (10). Surface markers like CD80 and CD86 are associated with the M1 phenotype, while CD23, CD163, and CD200R are linked to M2 macrophages (11).

Given the critical role of macrophages in host defense, some pathogens have evolved strategies to subvert the macrophage differentiation program by altering the M1 and M2 subtype commitment in their favor. For example, HIV-1, HCMV, and HCV benefit from M2 polarization (12–14), while an excessive M1 response can exacerbate diseases like H5N1 influenza and COVID-19 (15, 16). Certain viruses, such as PCV2, and bacteria like Listeria monocytogenes, are controlled by protective M1 polarization (17–19). *Salmonella Typhimurium* actively promotes an M2-like phenotype via its SPI-2 effector proteins to enhance intracellular replication (20). Nevertheless, the polarization states of macrophages following infection with key equine pathogens, including equine arteritis virus (EAV), equine herpes virus 1 (EHV-1), and equine infectious anemia virus (EIAV), remain poorly characterized, representing a significant gap in current research.

Macrophages from healthy horses can be activated either with IFN-γ + LPS or IL-4 to characterize M1- or M2-polarized macrophage gene expression (21–23). Other studies have suggested that equine macrophage polarization plays a vital role during repair in injured connective tissue (24). However, due to the lack of antibodies against equine macrophage polarization markers and the complicated methods required for the identification of equine macrophage polarization, the polarization phenotype of equine peripheral blood macrophages after infection is rarely reported. Because it is necessary to identify the polarization of equine macrophages, a simple and practical method to do so is necessary. CD80 and CD86 have often been used as M1 macrophage markers (25, 26). CD80 is a member of the extensively studied B7 class of transmembrane proteins, which can activate effective cellular immune responses. CD80 is primarily expressed on activated antigen-presenting cells such as macrophages, and its natural ligands are the T cell surface molecule CD28 and cytotoxic T-lymphocyte associated protein 4 (CTLA4, CD152) (27, 28). Under normal physiological conditions, the B7 family regulates the balance of the immune response. However, this coordination can be changed under pathological conditions (29). CD163 is a transmembrane protein and is a high-affinity scavenger receptor. It consists of eight scavenger receptor cysteine-rich (SRCR1 -8) regions and can recognize virus ligands (30–32). In undifferentiated monocytes, the expression of CD163 is very low; however, the expression of CD163 increases with activation and maturation of the monocytes (33). In humans, pigs, rodents, and other mammals, it has been confirmed that the CD163 molecule is mainly expressed on the surface of macrophages and can be used as an M2-specific marker (34, 35).

The purpose of this study was to develop a macrophage polarization detection system by generating monoclonal antibodies specific to equine M1-like and M2-like macrophage markers CD80 and CD163. To do this, we established a macrophage polarization model using equine macrophages stimulated with LPS and IFN-γ or IL-4 to induce M1-like or M2-like phenotypic changes, respectively. The M1-like phenotype was identified by the expression of TNF-α, IL-1β, IL-12, and CD80, whereas the M2-like phenotype was characterized by the expression of TGF-β, IL-10, CD206, and CD163. Furthermore, we present evidence that EIAV can induce circulating monocytes to differentiate into macrophages with M2 cytokine production, and the results from western blotting and flow cytometry experiments indicate elevated expression of M2 surface markers. Moreover, EHV-1, EAV, and *S. abortus equi* infections can effectively promote the release of M1 cytokines and upregulate the expression of M1 surface markers, suggesting that the polarization phenotype of equine macrophages could be used instead of the established methods of detection.

## RESULTS

### The morphology of equine macrophages exhibiting distinct marker profiles

**Fig 1:**
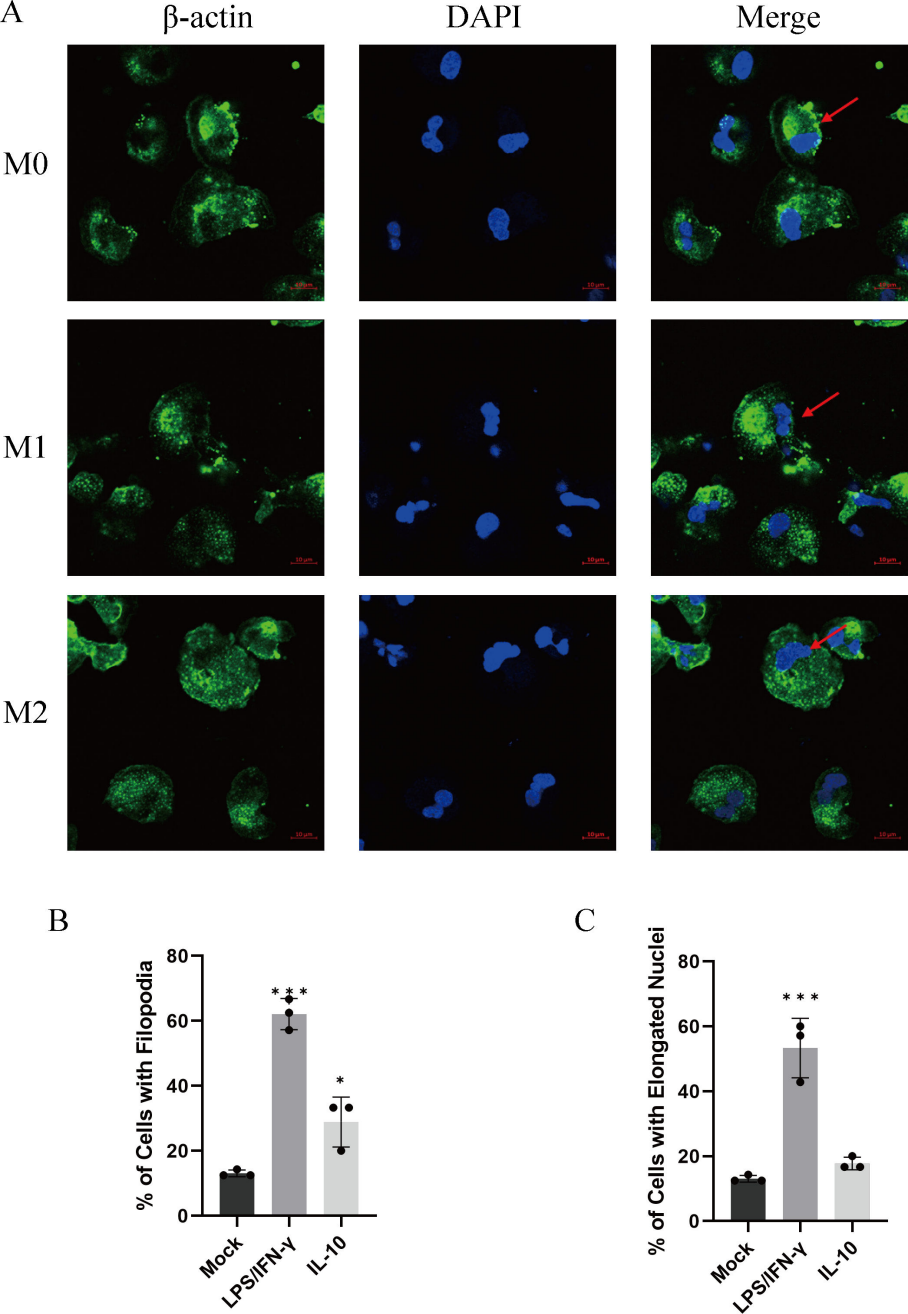
Analysis of macrophage morphology following different activation methods. (**A**) Equine macrophages were incubated for 24 h in RPMI medium in the presence of IFN-γ/LPS or IL-10. Cells were then fixed and labeled using the primary antibody β-actin and the secondary antibody Alexa Fluor 488. Nuclei were detected with DAPI (blue). The morphology of the control (M0), (M1) IFN-γ/LPS-, and (M2) IL-10-treated equine macrophages was examined with confocal microscopy. (**B**) Statistical analysis of filopodia-bearing macrophages post-induction. (**C**) Statistical analysis of the percentage of cells with elongated nuclei. Data are presented as mean ± SD (*n* = 3). *P < 0.05, ***P < 0.001 vs. Mock (M0) control.

Macrophage polarization, the process by which macrophages adapt their function in response to environmental cues, is associated with distinct morphological changes. While not definitive on their own, these morphological changes serve as supportive, secondary evidence to help classify macrophage phenotypic trends alongside primary molecular markers and cytokine profiles. Macrophages exhibit different degrees of elongation when stimulated with cytokines *in vitro* toward M1-like or M2-like phenotypes. Using a micropatterning approach to control the shape of cells directly, elongation of cells induces polarization toward an M2 phenotype. Moreover, elongation enhances the effect of M2-inducing cytokines and inhibits the effect of M1-inducing cytokines, suggesting that cell shape plays an important role in the modulation of the phenotypic polarization of macrophages (36, 37). Our investigation focused on elucidating morphological polarization patterns in equine macrophages. Notably, comparative analyses revealed distinct morphological differences between stimulated and control (M0) equine macrophages following 48-h treatments. Confocal imaging of macrophages stimulated with IFN-γ/LPS (M1-like) or IL-10 (M2-like) revealed that both subtypes extended numerous filopodial projections. Qualitative assessment indicated that M1-like macrophages displayed a markedly higher density of filopodia than M2-like cells (Fig. 1A).

M1 macrophages exhibited a significantly higher frequency of filopodia formation compared to the M0 and M2 groups (Fig. 1B). Additionally, M1 polarization was associated with marked nuclear morphology changes; a significantly higher percentage of M1-like cells displayed elongated nuclei compared to M0 and M2 cells, which retained oval or round nuclei (Fig. 1C). These morphological observations support the established phenotypic profiles reported in other species, in which filopodia enrichment correlates quantitatively with enhanced migratory capacity in pro-inflammatory M1 macrophages (37). However, these findings require further validation through quantitative analysis of molecular markers.

### M1-like/M2-like gene expression dynamics in stimulated equine macrophages

**Fig 2:**
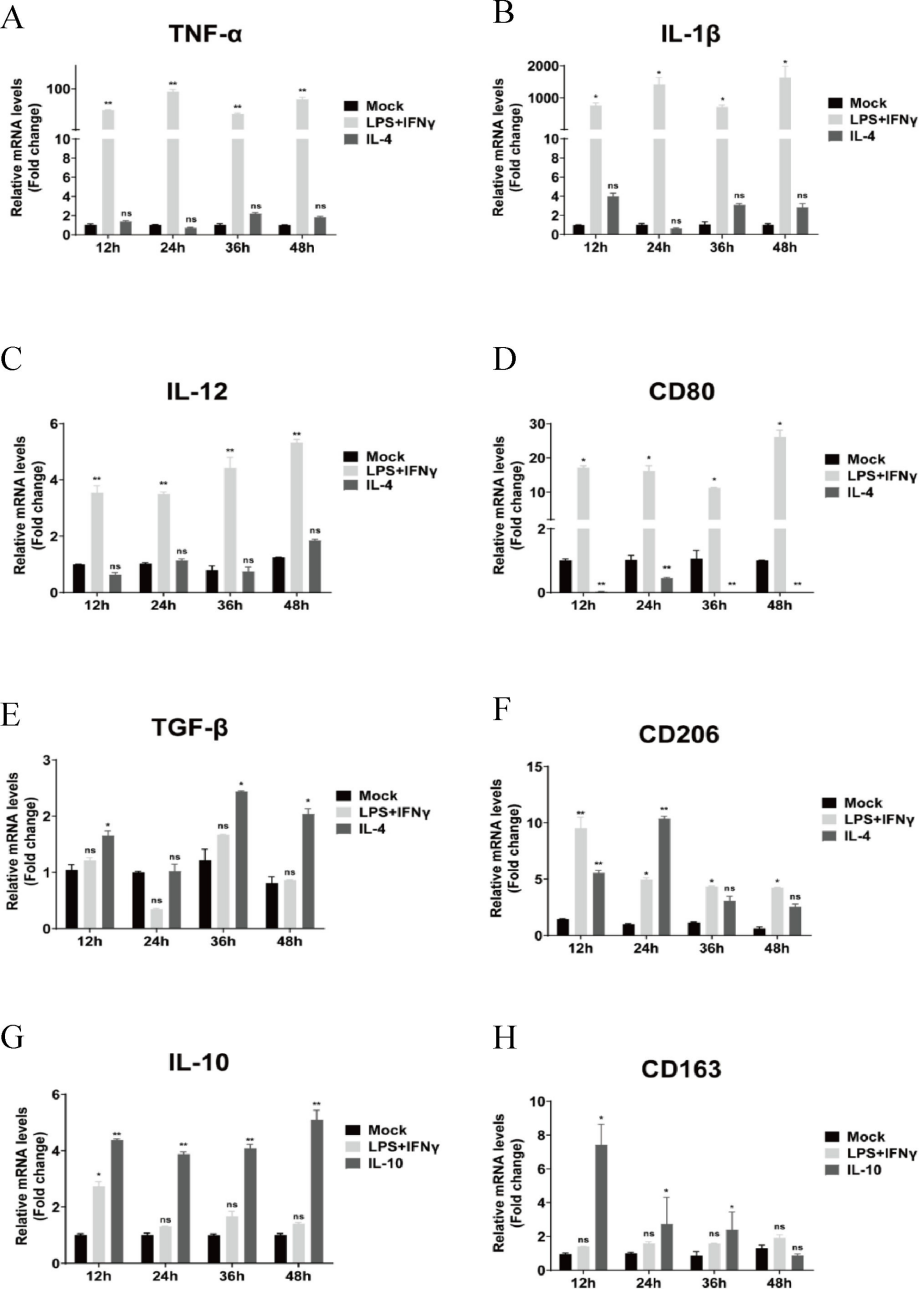
qRT-PCR analysis to determine optimal stimulation procedure for macrophage polarization. mRNA levels of genes of M1- and M2-type macrophages were monitored using qRT-PCR analysis following treatment with various concentrations of LPS/IFN-γ, IL-4, or IL-10, respectively. (**A–D**) Figure panels show the expression of M1-type macrophage polarization markers, such as TNF-α, IL-1β, IL-12, and CD80, after 12, 24, 36, and 48 h of incubation. (**E–H**) Figure panels show the distinct expression profiles of M2-like markers, specifically the IL-4-induced upregulation of TGF-β (**E**) and CD206 (**F**), and the IL-10-induced upregulation of IL-10 (**G**) and CD163 (**H**), after 12, 24, 36, and 48 h of incubation. Data represent the mean ± standard error of the mean (SEM) of at least three independent experiments.

The morphological changes in equine macrophages in different polarization states suggest that the two different polarized macrophages have different gene expression profiles. While M1/M2 activation signatures are well-documented in murine and human models, their gene transcriptional regulation in equines required systematic validation. For M1-like phenotypic induction, macrophages were exposed to a combination of 1,000 ng/mL LPS and 100 ng/mL IFN-γ. Quantitative analysis at sequential time points (12, 24, 36, and 48 h) revealed progressive upregulation of canonical M1 markers: TNF-α exhibited a 65-fold increase by 12 h (*P* < 0.01) (Fig. 2A), while IL-1β and IL-12 reached peak expression at 48 h (1008.2-fold and 5.4-fold, respectively; *P* < 0.01) (Fig. 2B and C). The surface marker CD80 showed elevated levels, achieving statistical significance (*P* < 0.01) from 12 h to 48 h (Fig. 2D). Simultaneously, the expression of NO and NOS2 was significantly increased in macrophages following treatment with LPS/IFN-γ (*P* < 0.01) (Fig. S1A through C). Parallel experiments using 100 ng/mL IL-4 or IL-10 demonstrated distinct M2-like kinetic programs specific to each inducer. TGF-β mRNA levels increased 3.1-fold within 36 h (*P* < 0.05) under IL-4 stimulation (Fig. 2E). Treatment with IL-4 induced a time-dependent upregulation of the scavenger receptor CD206, peaking at 24 h with a 7.4-fold increase (*P* < 0.01) (Fig. 2F). Contrasting with IL-10-induced macrophages where autocrine IL-10 production peaked later at 48 h (5.8-fold; *P* < 0.01) (Fig. 2G). CD163 expression was significantly enhanced by IL-10 in a time-dependent manner, reaching a maximum 5.9-fold increase at 12 h (*P* < 0.05) (Fig. 2H). While CD206 is a canonical M2 marker, we observed unexpected upregulation following LPS/IFN-γ treatment, confounding its utility as a discriminator. Conversely, CD163 displayed a distinct expression profile restricted to IL-10 stimulation, highlighting the phenotypic divergence between IL-4- and IL-10-induced M2-like states. Therefore, to ensure phenotypic specificity, CD163 was selected as the core marker for the IL-10-driven M2-like profile in further analyses. These results suggest that M1 macrophages also express high levels of surface markers such as CD80, while M2 macrophages also express high levels of surface markers such as CD163.

### Development and validation of monoclonal antibodies targeting CD80 and CD163

**Fig 3:**
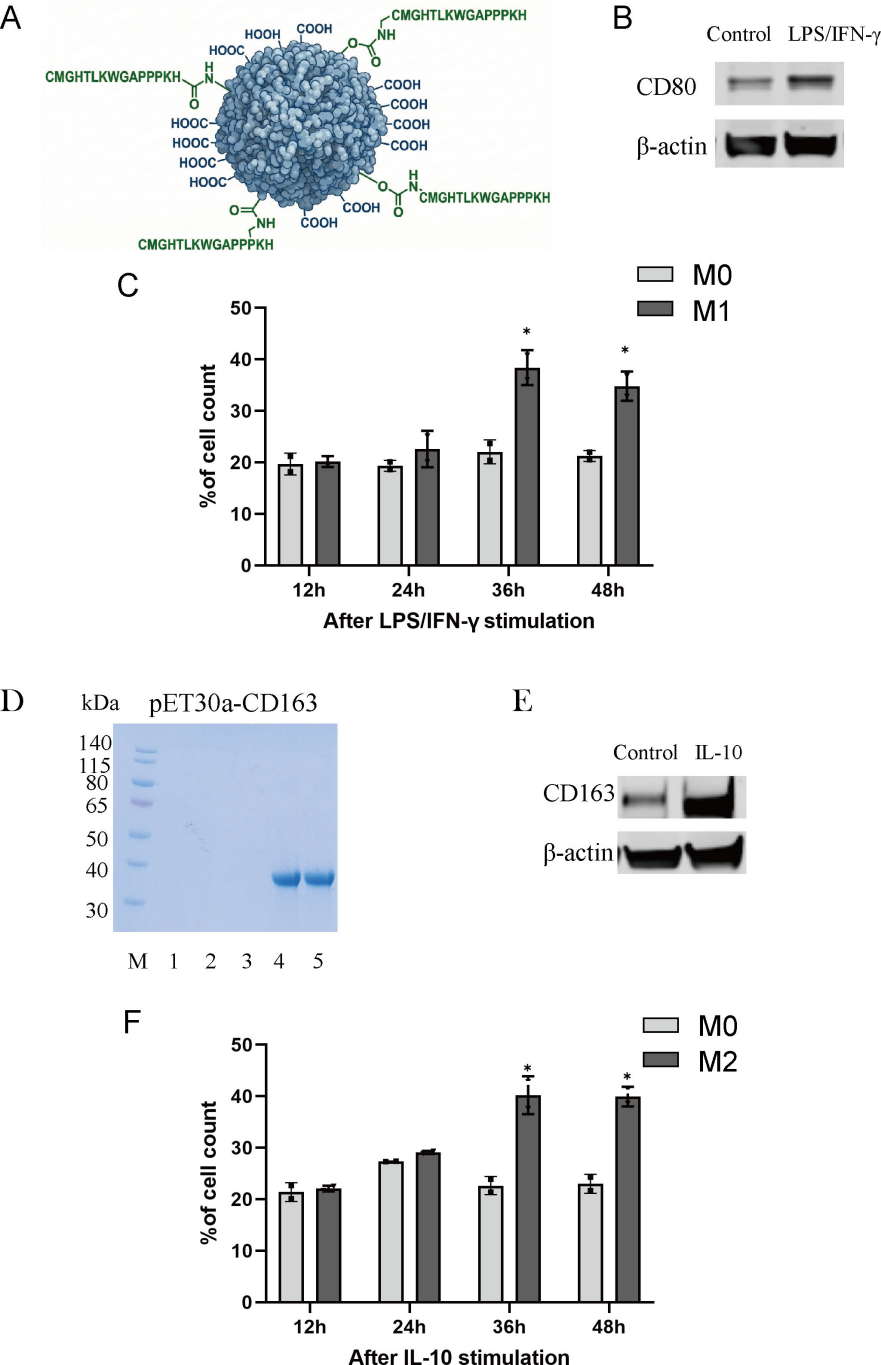
Development and validation of monoclonal antibodies targeting CD80 and CD163. (**A**) Schematic illustration of the conjugation of synthetic CD80 peptides to Keyhole Limpet Hemocyanin (KLH). (**B**) Western blot detection of endogenous CD80 protein in LPS/IFN-γ-stimulated macrophages using an anti-CD80 monoclonal antibody. (**C**) Flow cytometry analysis was performed to determine the proportion of CD80-positive cells after LPS/IFN-γ treatment for 12, 24, 36, and 48 h. (**D**) Expression and purification of CD163 protein; SDS-PAGE analysis of purified recombinant CD163 using a 12% acrylamide gel. Lanes: M, protein markers; 1–3, the bacterial pellets after lysis and centrifugation; 4–5, purified protein of pET-30a-CD163. (**E**) Western blot analysis was performed to detect the expression of endogenous CD163 using a CD163 antibody after IL-10 treatment of cells for 24 h. (**F**) Flow cytometry analysis was performed to determine the proportion of CD163-positive cells after IL-10 treatment for 12, 24, 36, and 48 h.

To enable precise discrimination between M1-like and M2-like equine macrophages, monoclonal antibodies (mAbs) against the surface markers CD80 and CD163 were developed. This study was initiated based on our preliminary findings: CD80 exhibited significant upregulation in M1 macrophages, while CD163 demonstrated more pronounced elevation in IL-10-driven M2-like populations compared to the IL-4-induced marker CD206 (Fig. 2). Due to the presence of two transmembrane domains in CD80 that hinder its expression and purification, the extracellular domain of CD80 was represented by a synthetic 15-amino acid peptide conjugated to carrier proteins (Fig. 3A). The schematic workflow for monoclonal antibody preparation is illustrated in (Fig. S1D). Following immunization of BALB/c mice with the CD80 peptide, serum titers were monitored, and spleen cell fusion was carried out upon achievement of a high titer (Fig. S1E). Hybridoma supernatants were screened via ELISA and western blot, identifying the stable anti-CD80 clone 5C4. Specificity validation of the CD80 antibody by western blot analysis in human (HEK293 and THP-1) and murine (RAW 264.7) cell lines demonstrated that it only recognizes equine macrophages (Fig. S3A). Subsequent western blot analysis using this monoclonal antibody confirmed significant upregulation of CD80 expression in macrophages following LPS/IFN-γ stimulation (Fig. 3B; Fig. S1G). A secondary antibody-only control was used to establish baseline fluorescence and gating. The proportion of CD80-positive macrophages was markedly reduced following transfection with siCD80 compared to the negative control (siNC) (Fig. S3C and E). Flow cytometry was used to validate the proportion of CD80-positive cells in macrophages following LPS/IFN-γ stimulation. The cells were analyzed at 12, 24, 36, and 48 h post-stimulation, revealing a significant increase in the percentage of CD80-positive cells after 36 h compared to the control group (Fig. 3C; Fig. S2A).

For CD163, a truncated version lacking transmembrane domains was expressed in *E. coli* BL21 using a prokaryotic system, followed by affinity purification. SDS-PAGE confirmed successful expression, with distinct bands at 36 kDa (Fig. 3D). BALB/c mice underwent three rounds of immunization with the purified CD163 protein. Serum antibody titers were quantified with ELISA, with high-titer individuals selected for hybridoma generation (Fig. S1F). Splenocytes from immunized mice were fused with immunoglobulin-non-secreting myeloma cells using standard hybridoma techniques (38). Supernatants from microplate cultures were screened for antigen-specific binding through sequential ELISA and western blotting, and the highly reactive anti-CD163 clone, 1C6, was successfully isolated. Western blot analysis of human (HEK293 and THP-1) and murine (RAW 264.7) lysates confirmed the high specificity of the antibody for the equine ortholog, as no signal was detected in non-equine lines (Fig. S3B). Subsequent western blot analysis using this monoclonal antibody confirmed significant upregulation of CD163 expression in macrophages following IL-10 stimulation (Fig. 3E; Fig. S1H). A secondary antibody-only control was used to establish baseline fluorescence and gating. The proportion of CD163-positive macrophages was markedly reduced following transfection with siCD163 compared to the negative control (siNC) (Fig. S3D and F). Flow cytometric analysis confirmed a time-dependent induction of CD163 by IL-10, with the percentage of CD163-positive cells significantly increasing at 36 h compared to untreated controls (Fig. 3F; Fig. S2B). Confocal microscopy of macrophages co-stained for CD14 (pan-macrophage marker) and CD80/CD163 demonstrated co-localization, verifying the expression of CD80/CD163 on the macrophage surface (Fig. S4A and B). These results indicate that the prepared CD80 and CD163 antibodies are suitable for subsequent studies on macrophage polarization following pathogen infection.

### Analysis of pathogen infection of equine macrophages

**Fig 4:**
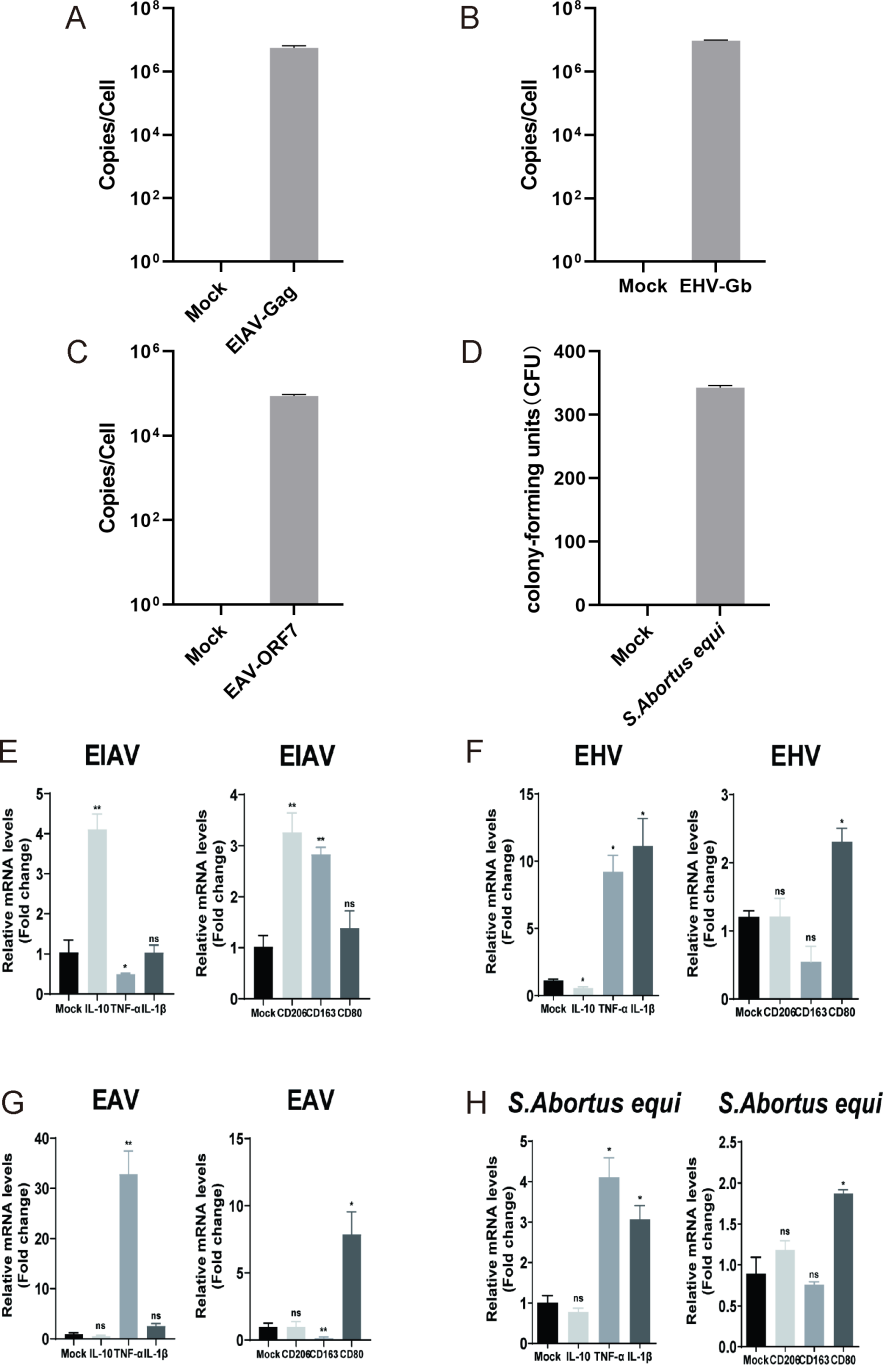
Assessment of pathogen replication and qRT-PCR analysis of polarization changes in macrophages following bacterial and viral infection. (**A**) Measurement of viral copy number in EIAV-infected macrophages using quantitative real-time PCR. (**B**) Measurement of viral copy number in EHV-1-infected macrophages using quantitative real-time PCR. (**C**) Measurement of viral copy number in EAV-infected macrophages using quantitative real-time PCR. (**D**) Colony counting of *S. abortus equi*-infected macrophages. (**E**) mRNA levels of cytokines and surface markers after EIAV infection. (**F**) mRNA levels of cytokines and surface markers after EHV-1 infection. (**G**) mRNA levels of cytokines and surface markers after EAV infection. (**H**) mRNA levels of cytokines and surface markers after *S. abortus equi* infection.

In this study, we verified the expression of macrophage polarization markers and successfully generated monoclonal antibodies targeting specific surface markers. These methodologies enable precise identification of equine macrophage polarization status post-pathogen infection, while the developed antibodies serve as essential biological tools for elucidating mechanisms underlying pathogen-host interactions. To ensure effective pathogen infection of the equine macrophages, we assessed the infection models. For EIAV, viral load was quantified 24 h post-infection, revealing target gene copy numbers exceeding 10^6^ copies/cell (Fig. 4A). For EHV-1, similar quantification at 24 h post-infection also demonstrated target gene copy numbers above 10^6^ copies/cell (Fig. 4B). For EAV, the results showed target gene copy numbers surpassing 10⁴ copies/cell at the same time point (Fig. 4C). Furthermore, for *S. abortus equi*, significantly more bacterial colonies were observed on plates cultured with infected cell lysates compared to the negative control group (Fig. 4D). Collectively, these results confirm that the pathogens were effectively internalized by the equine macrophages.

### qPCR analysis of phenotypic marker profiles of equine macrophages following pathogen infection

Different pathogen infections can cause macrophages to develop different polarization phenotypes. Following the confirmation of successful pathogen infection, qPCR analysis was conducted to measure mRNA expression levels of six polarization-related genes in equine macrophages. The results showed that different pathogen infections can lead to different polarization of equine macrophages. After EIAV infection, IL-10, CD206, and CD163 exhibited varying degrees of upregulation, suggesting macrophage polarization toward an anti-inflammatory M2-like phenotype to modulate inflammatory responses (Fig. 4E). Conversely, infections with EHV-1, EAV, and *S. abortus equi* triggered significant elevation of TNF-α, IL-1β, and CD80, indicating predominant M1-like marker profiles and subsequent release of pro-inflammatory mediators during initial infection phases (Fig. 4F through H).

### Flow cytometry and western blot analysis of phenotypic changes of equine macrophages after pathogen infection

**Fig 5:**
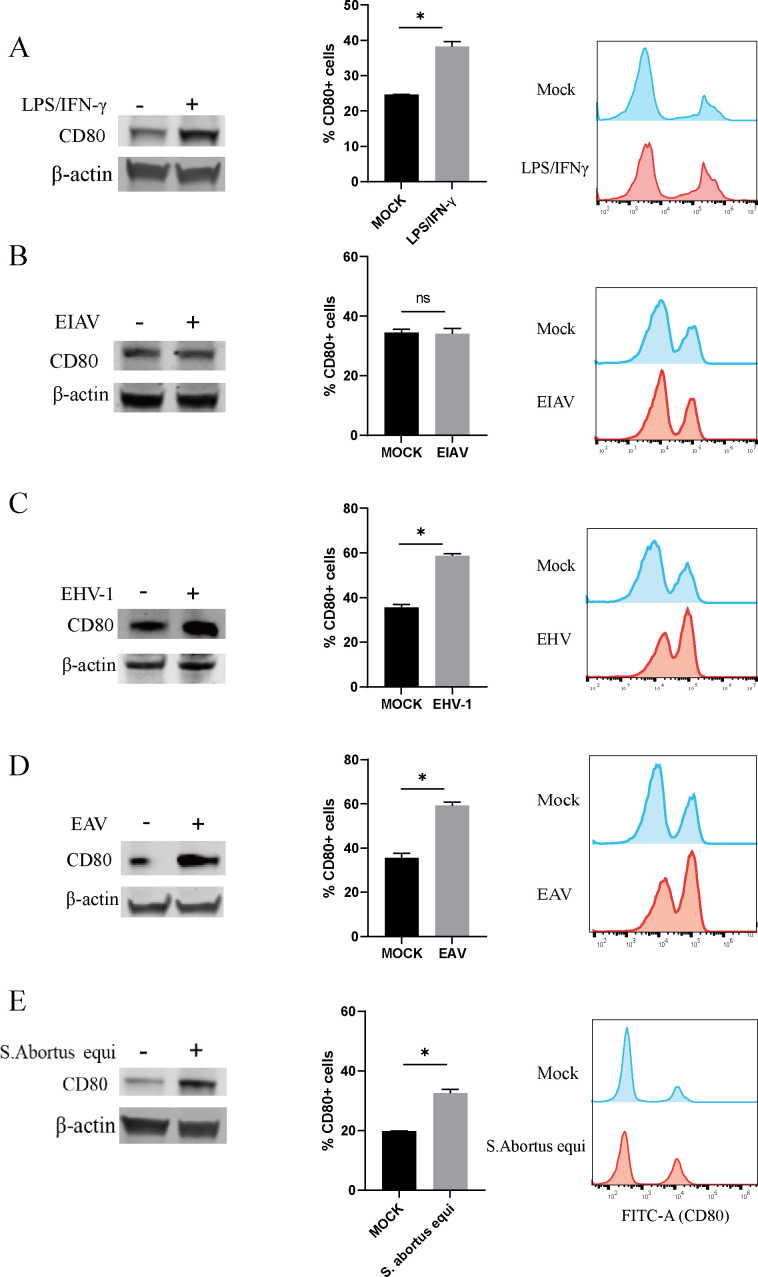
Analysis of CD80 expression in pathogen-infected equine macrophages. CD80 protein levels and the percentage of CD80-positive cells were quantified using Western blotting (left panels) and flow cytometry (right panels), respectively. The “mock” columns represent uninfected, unstimulated equine macrophages serving as the negative baseline control. (**A**) Positive control demonstrating CD80 upregulation following LPS/IFN-γ stimulation. (**B–E**) CD80 expression profiles following infection with (**B**) EIAV, (**C**) EHV-1, (**D**) EAV, and (**E**) *S. abortus equi*. For flow cytometry histograms, the x-axis represents FITC-fluorescence intensity (CD80 expression), and the y-axis represents cell count. Bar graphs present the mean ± SEM of three independent biological replicates. Statistical significance relative to the mock control was determined using a Student’s *t*-test (*, *P* < 0.05; **, *P* < 0.01; ***, *P* < 0.001).

**Fig 6:**
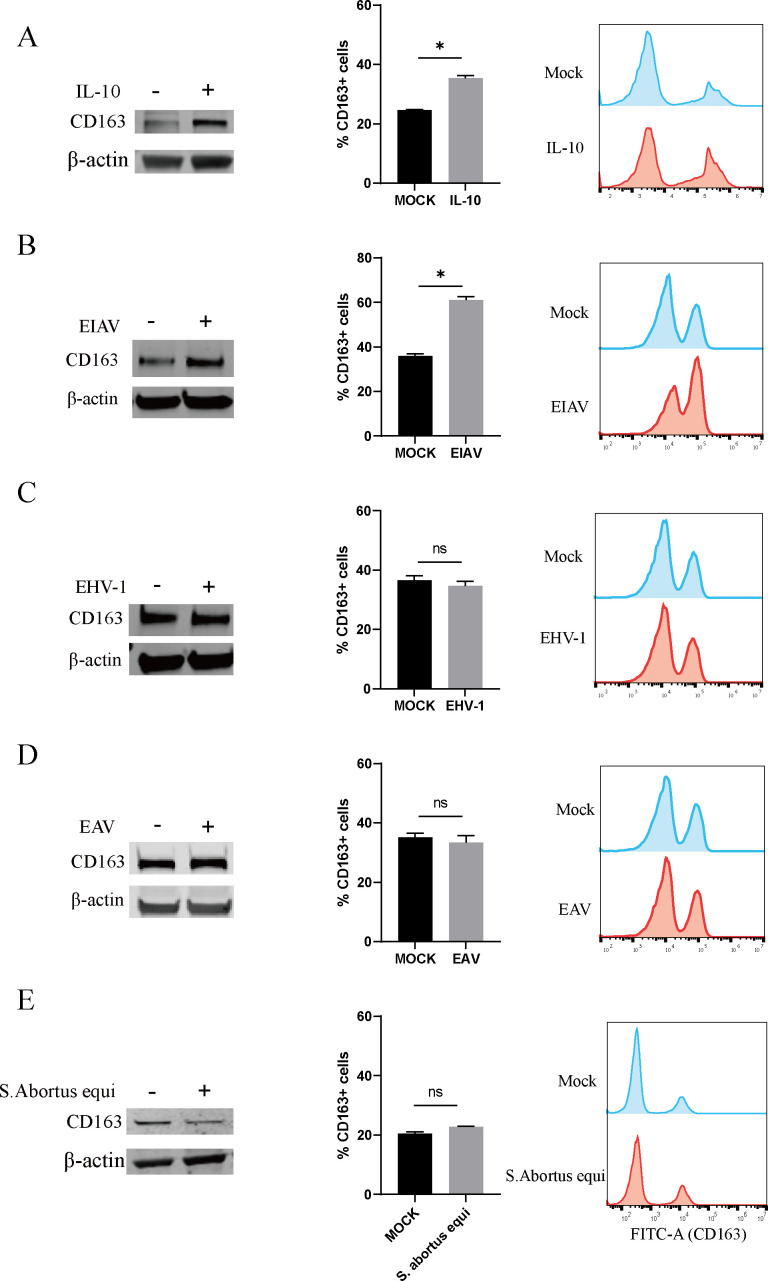
Analysis of CD163 expression in pathogen-infected equine macrophages. CD163 protein levels and the percentage of CD163-positive cells were quantified using Western blotting (left panels) and flow cytometry (right panels), respectively. The “mock” columns denote uninfected, unstimulated negative control cells. (**A**) Positive control demonstrating CD163 upregulation following IL-10 stimulation. (**B–E**) CD163 expression profiles following infection with (**B**) EIAV, (**C**) EHV-1, (**D**) EAV, and (**E**) *S. abortus equi*. Flow cytometry histograms display FITC-fluorescence intensity (CD163 expression) on the x-axis and cell count on the y-axis. Data are shown as mean ± SEM of three independent biological replicates. Statistical differences relative to the mock control were assessed via Student’s *t*-test (*, *P* < 0.05; **, *P* < 0.01; ***, *P* < 0.001).

Flow cytometry, a high-resolution analytical technique for single-cell quantification, and western blot analysis, a gold-standard method for total protein detection, were synergistically employed to investigate pathogen-induced macrophage polarization (39, 40). The combined methodology revealed distinct polarization patterns. To characterize the marker profiles induced by different pathogens, we analyzed the expression of the M1 marker CD80 (Fig. 5) and the M2 marker CD163 (Fig. 6) using both western blot and flow cytometry. Regarding the validation of polarization markers, positive control treatments performed as expected and validated the assay specificity. Specifically, stimulation with LPS/IFN-γ (M1 inducer) significantly upregulated CD80 expression (Fig. 5A), while treatment with IL-10 (M2 inducer) significantly enhanced CD163 levels (Fig. 6A).

Regarding pathogen-induced polarization, EIAV infection elicited a distinct M2-like phenotype. Both flow cytometry and western blot analysis revealed a significant upregulation of the M2 marker CD163 (Fig. 6B). In contrast, CD80 expression in EIAV-infected cells remained comparable to mock-infected controls (Fig. 5B).

Conversely, infections with EHV-1, EAV, and *S. abortus equi* exhibited an M1-dominant profile. All three pathogens induced a significant increase in the proportion of CD80-positive cells and total CD80 protein expression (Fig. 5B, C and E). However, unlike the EIAV group, CD163 expression in these groups did not differ significantly from the mock control (Fig. 6B, D and E).

Collectively, these data suggest that while EIAV drives macrophage polarization toward an M2-like phenotype, EAV, EHV-1, and *S. abortus equi* preferentially induce M1-like marker profiles.

## DISCUSSION

Monocytes and monocyte-derived macrophages are central effector cells of the host innate immune response against diverse pathogens, and their roles have been extensively characterized in viral and bacterial infectious diseases. Macrophage polarization represents a highly dynamic and multifaceted immunological process, with distinct subpopulations displaying unique molecular mediator expression and secretion profiles. Considerable effort has been devoted to identifying biomarkers that distinguish human M1-like and M2-like macrophage subsets, which are invaluable for defining cellular activation states and functional contributions to inflammatory pathophysiology (6). While macrophage polarization has been well characterized in mice and humans, validated, antibody-based standardized protocols for quantifying equine macrophage M1-like and M2-like biomarkers remain unavailable (21–23). Previous studies have relied on metabolic profiles or cytokine secretion to indirectly identify equine macrophage polarization phenotypes (24). Thus, comprehensive characterization of equine macrophage subsets—through integrated morphological, surface marker, and cytokine profiling—is essential to establish precise phenotypic definitions for these cells in horses.

Morphological adaptations provide the first line of evidence for this phenotypic shift. Stimulation of equine macrophages with LPS/IFN-γ induced an elongated cellular morphology, whereas IL-10 treatment promoted a rounded phenotype (Fig. 1A). These observations indicate that macrophages exhibiting an M1-like profile enhance their phagocytic capacity via cytoskeleton-driven morphological remodeling, consistent with findings in human macrophage systems where activation protocols fundamentally reshape cellular architecture and function (36).

Beyond morphology, our comparative analysis validated distinct biochemical profiles for these subsets. We observed significantly higher production of IL-1β, TNF-α, IL-12, and CD80 in LPS/IFN-γ-activated equine macrophages compared to IL-10-treated cells (Fig. 2A through D). Conversely, equine M2-like macrophages were defined by elevated expression of TGF-β, IL-10, CD206, and CD163 (Fig. 2E through H). These results closely align with the canonical marker profiles (such as CD86, iNOS, and TNF-α for M1; Arginase-1 and CD206 for M2) established in polarized human macrophages. Because CD80 and CD163 mark discrete functional states—with CD80 labeling pro-inflammatory M1-like macrophages and CD163 identifying anti-inflammatory M2-like macrophages—they were selected as the core phenotypic biomarkers for this study.

To operationalize these markers, we generated monoclonal antibodies against equine CD80 and CD163 using antigen designs tailored to overcome technical hurdles in hybridoma development. For CD80, the short extracellular domain limited immunogenicity, prompting the use of carrier-conjugated synthetic peptides to boost immune responses (Fig. 3A). For CD163, the extracellular region was selected as the immunogen based on bioinformatic epitope prediction and cross-species structural conservation. CD80, a transmembrane protein essential for T-cell activation via CD28 and CTLA-4 interactions (27), has been validated as a reliable M1 marker across humans, mice, pigs, and cattle. Its upregulation is fundamentally tied to the antimicrobial and antitumor defenses of M1 macrophages. Similarly, CD163, a scavenger receptor mediating anti-inflammatory responses and tissue repair, has been universally confirmed as a specific M2 phenotypic marker across mammalian species (31–35).

Ultimately, equine CD80 and CD163 represent reliable surface markers for the *in vitro* classification of macrophage phenotypic states. Specifically, CD80 identifies an M1-like marker profile induced by IFN-γ and LPS, while CD163 marks M2-like phenotypes driven by IL-10. This approach provides a standardized, reproducible tool for preliminary screening and mechanistic pathway investigations.

Nevertheless, several limitations warrant careful consideration. First, the absence of isolated LPS-only and IFN-γ-only controls in our experimental design prevents us from definitively disentangling the individual contributions of these stimuli to the observed M1-like profile. Second, the use of IL-4 and IL-10 as M2-like inducing stimuli represents a simplified *in vitro* model that may elicit a hybrid activation state rather than fully recapitulating the discrete M2a, M2b, and M2c subsets observed *in vivo* (10). Additionally, conventional two-dimensional (2D) monoculture systems fail to recapitulate the three-dimensional tissue architecture and dynamic cell-cell interactions required for physiologically relevant macrophage polarization. This limitation is underscored by reports of altered marker expression profiles in co-culture environments (41, 42). Moreover, the observed co-expression of CD80 and CD163 under certain pathological conditions reflects the inherent plasticity of macrophages and challenges the rigid binary M1/M2 polarization paradigm (43). Our findings should therefore be corroborated using expanded marker panels and more physiologically relevant models, such as three-dimensional (3D) co-culture systems, to enhance the translational potential of our results. Future studies should prioritize the identification and functional characterization of distinct equine M2 subsets to refine our understanding of equine immunology and facilitate the development of targeted therapeutic strategies.

Building upon these *in vitro* models, we applied our established multi-modal approach (qPCR, western blotting, and flow cytometry) to examine phenotypic changes following infection with diverse equine pathogens. Both western blotting (providing precise total protein quantification) and flow cytometry (enabling high-resolution population characterization) were critical for this analysis (39, 40), particularly to overcome the limitations of immunocytochemical approaches that often struggle to distinguish macrophages from functionally similar dendritic cells (44).

Our findings revealed highly pathogen-specific immune regulatory programs. Following infection with the attenuated EIAV-DLV121 vaccine strain, M2-like markers (CD163 and CD206) were upregulated, while M1-associated cytokines (TNF-α and IL-1β) were reduced (Fig. 4E). Flow cytometry and western blotting confirmed this CD163 upregulation occurred without altering CD80 expression (Fig. 5B and 6B). These observations align with our previous reports (45), which demonstrated that the pathogenic EIAV strain EIAV-DLV34 drives macrophages toward an M1-like phenotype instead, revealing a phenotypic switch intrinsically linked to viral virulence. This strain-dependent divergence is likely mediated by the differential regulation of key signaling pathways, including the JAK/STAT axis. Consequently, a critical limitation of our current study is that the observed promotion of an M2-like marker profile was induced specifically by an attenuated vaccine strain. These findings must not be broadly generalized to all EIAV infections, particularly those caused by wild-type or field strains. Future *in vivo* studies utilizing pathogenic clinical isolates are necessary to fully elucidate the complexities of EIAV-macrophage interactions during natural infection.

In contrast, infection with EHV-1, EAV, or *S. abortus equi* downregulated M2 markers and increased M1 cytokine expression (Fig. 4F through H), upregulating CD80 without affecting CD163 levels (Fig. 5C through E and 6C through E). The strong M1-like marker profile induced by pathogenic strains such as EHV-1 and EAV resembles patterns seen in severe infections like SARS-CoV and influenza, where early inflammatory responses are critical for pathogen control but can also drive immunopathology (15, 16, 46). Conversely, the M2-like skewed profile elicited by the attenuated EIAV-DLV121 strain likely represents an immunomodulatory strategy to limit inflammatory damage, a mechanism shared by persistent viruses such as human cytomegalovirus (HCMV) (47).

Furthermore, the distinct metabolic profiles observed in these subsets provide a functional basis for their polarization states. Metabolic reprogramming directly supports subset-specific effector functions: glycolysis enables the rapid antimicrobial responses characteristic of M1-like macrophages, while reliance on the tricarboxylic acid cycle with slower oxidative phosphorylation sustains the tissue-reparative and immunomodulatory functions of M2-like cells (48). Ultimately, the divergent macrophage polarization phenotypes induced by these equine pathogens provide a vital mechanistic framework for understanding their distinct pathogenic outcomes: acute inflammatory injury versus attenuated, non-pathogenic persistence.

In conclusion, the macrophage marker profile detection method established in this study enables rapid identification of *in vitro* macrophage phenotypic states and holds strong potential for drug discovery, toxicological screening, and immunotherapy evaluation. This approach can be broadly applied to study macrophage phenotypic changes in equine infectious diseases. A deeper understanding of pathogen-driven macrophage phenotypic changes will support the development of more effective antimicrobial interventions and vaccines for equine species.

## MATERIALS AND METHODS

### Bacteria, viruses, and cells

*S. abortus equi* and EIAV were stored in our lab, *Equid herpesvirus-1* (EHV-1) was isolated from infected tissue samples through cell culture propagation and stored in our lab, and EAV strain (Bucyrus strain) was kindly provided by Professor Zhang Nianzu from the Yunnan Tropical and Subtropical Animal Viral Disease Laboratory.

Preparations of eMDMs were obtained from equine peripheral blood mononuclear cells (PBMCs) as described previously, with a minor modification (49). Briefly, PBMCs were isolated from 200 to 300 mL of heparinized horse peripheral blood by centrifugation through a HybriMax Histopaque cushion (density = 1.077 g/cm^3^; Sigma, USA). Isolated PBMCs were washed with RPMI 1640 medium three times and resuspended in RPMI 1640 medium supplemented with 10% horse serum, 10^4^ U/mL penicillin, 10^4^ μg/mL streptomycin, 2 mM L-glutamine, 0.1 mM nonessential amino acids, 1 mM sodium pyruvate, and 0.25 mM sodium HEPES. These cells were seeded into tissue culture flasks (Corning, USA) at 5 × 10^6^ cells/25 cm^2^ and incubated at 37°C in 5% CO_2_ for approximately 12 h. Nonadherent and loosely adherent cells were removed by mildly shaking the flasks before changing the medium, and the remaining adherent cells were further incubated for 3 days to allow differentiation into eMDMs. Murine SP2/0 myeloma cells kept in our laboratory were cultured in RPMI-1640 medium with 10% FBS (Ausbian). Hybridoma cells were cultured in the above medium containing HAT (Sigma). The positive hybridoma cells were cultured in the above medium containing HT (Sigma).

### Confocal microscopy

Equine macrophages were treated either with M1 inducer (LPS, final concentration 1 μg/mL and IFN-γ, final concentration 100 ng/mL) or with M2 inducer (IL-10, final concentration 100 ng/mL). After 24 h of treatment, the macrophages were washed with cold PBS and fixed with 4% paraformaldehyde for 15 min at room temperature. The fixed cells were permeabilized with 0.1% Triton X-100 for 15 min before blocking in 5% skim milk for 2 h. The cells were incubated with the primary antibody (β-actin, 1/500, mouse, Sigma) at 4°C overnight. After three washes, the secondary antibodies coupled to a fluorochrome (Alexa Fluor 488 dye goat anti-mouse IgG) were added for 1 h. Samples were washed three times in PBS, and nuclei were labeled with DAPI (Beyotime, China). Images were acquired with a confocal microscope (LSM800; ZEISS, Germany).

### Preparation of monoclonal antibodies against CD80 and CD163

The CD80 polypeptide used to immunize mice was synthesized by Nanjing Genscript Biotech Co., Ltd. (Nanjing, China). According to the predicted antigen epitope distribution and the immunogenic region of human and mouse CD80, 15 amino acids at the N-terminal of the sequence (CMGHTLKWGAPPPKH) were conjugated with Ovalbumin (OVA) and KLH, respectively. SRCR1 ~SRCR4 fragments of CD163 were inserted into the plasmid pET-30a by in-fusion cloning to transform *E. coli* BL21 competent cells. CD163 recombinant protein was expressed and purified for the immunization of mice.

CD80 polypeptide or CD163 recombinant protein was mixed with an adjuvant in a ratio of 1:1 to immunize 6-week-old female BALB/c mice. Monoclonal antibody (mAb) was prepared using the hybridoma technique, and indirect ELISA was used to screen for positive hybridoma cells (50). The antibody secreted in the supernatant of the cell culture medium was used as a primary antibody, and an HRP-conjugated mouse secondary antibody was used. The positive hybridoma cells were then subjected to limited dilution to ensure they were monoclonal. Identification of the mAb isotype was carried out according to the instructions of the SBA Clonotyping system-HRP (Southern Biotech). Hybridoma cells with good specificity and IgG subtype were injected into the mouse abdominal cavity. One week after injection, the ascites fluid was collected and purified according to the HiTrap Protein G HP (GE) specification.

### siRNA design and transfection

To investigate the functional role of CD80 and CD163 in equine macrophages, gene silencing was performed using small-interfering RNAs (siRNAs) specifically targeting Equus caballus mRNA sequences. The specific siRNA duplexes were synthesized by Sangon Biotech (Shanghai, China). The sequences for the CD80 siRNA were: sense 5′-CCUUCACCGACAUCACCAA-3′ and antisense 5′-UUGGUGAUGUCGGUGAAGG-3′. The sequences for the CD163 siRNA (clone 146) were: sense 5′-GCUGGUAAAGAUAAUUGUA-3′ and antisense 5′-UACAAUUAUCUUUACCAGC-3′. A non-targeting scrambled siRNA was used as a negative control in all experiments. Transfection was carried out using the RNAmix transfection reagent (Invitrogen, Carlsbad, CA, USA) according to the manufacturer’s instructions. Briefly, equine macrophages were seeded into six-well plates. The siRNA duplexes and RNAmix reagent were diluted separately in Opti-MEM reduced-serum medium. The diluted siRNA and transfection reagent were then combined and incubated for 5 min at room temperature to allow complex formation. The transfection complexes were added dropwise to the cells to achieve a final siRNA concentration of 50 nM. Cells were incubated at 37°C in a 5% CO₂-humidified atmosphere for 36 h before harvesting for further analysis.

### Bacterial and viral infection of equine macrophages

Fresh *S. abortus equi* bacterial suspension (1 × 10^8^ CFU/mL) and EHV-1 (10^6^ PFU/mL), EAV (5 × 10^6^ TCID_50_/mL), and EIAV (5 × 10^5^ TCID_50_/mL) were used to infect equine macrophages. One group was washed and supplemented with a culture solution containing penicillin and streptomycin 2 h after each bottle was infected with 10 μL *S. abortus equi*. Macrophages that were not infected with bacteria were set as a negative control. The other three groups were infected with 100 μL EHV-1, 100 μL EAV, and 1 mL EIAV for 30 min, and fresh culture medium was supplemented afterward. Macrophages that were not infected with any virus were set as a negative control. In the bacterial cultures, after 12 h of bacterial infection, the cells were washed with PBS and then lysed, and the lysate was spread over plates for culture. For the virus-infected cells, after 24 h virus infection, TRIzol was added to extract total RNA of the cells and reverse-transcribed it into cDNA. Three virus-specific genes, EHV-Gb, EAV-ORF7, and EIAV-Gag, were detected using quantitative PCR (qPCR) and used for quantitative analysis of the virus in the cells.

### RNA extraction and qPCR

Equine macrophages were infected with pathogens as described above. Total RNA was then extracted from the cells with TRIzol (Invitrogen) according to the manufacturer’s instructions and was reverse-transcribed into cDNA. Transcription levels of eight genes (TNF-α, IL-1β, IL-12, TGF-β, IL-10, CD80, CD206, and CD163) were assessed using qPCR, and macrophages without infection were set as negative controls. qPCR was performed using SYBR Premix Ex Taq (Takara). Relative gene expression was evaluated using the 2^−ΔΔCt^ method (51). β-actin served as an endogenous control. The specific primers used are listed in Table 1.

**TABLE 1:** qRT-PCR primer sequences

Target gene	Primer sequence (5′−3′)	Product length/bp
TNF-α	F: ATGAGCACTGAAAGCATGATCCGAGATG	117
	R: GAGGAAGGAGAAGAGGCTGAGGCACAAG	
IL-1β	F: GCTTCAATTCTCCCACCAACTTTACAAC	150
	R: TCAAAGATGACAGAAAAGAGGCT	
IL-12	F: TGTGCCTTAGCAGCATCTATGAGGACTT	238
	R: GCATGAAGAAGGATGCAGAGCTTGACTT	
TGF-β	F: CCACTGCCCCTGTGACAGCAAAGATAAC	260
	R: AGCCCAGATCCTTGCGAAAGTCAATGTA	
IL-10	F: AAAATAAGAGCAAGGCAGTGGAGCAGGTG	115
	R: TAGGCTTCTATGTAGTTGATGAAGATGTCA	
CD80	F: ACGGAACAGAAGTAAATGCCATCAACAC	214
	R: GTCAGGAGCGGGGACTGGTTAGTAGGAG	
CD206	F: AGGACAAACACCAAAACCTGAGCCAACA	309
	R: GCCACATAATCCACTTTGCTTCCATCCA	
CD163	F: GTGTTCAGGCAGAATAGAGGTCA	285
	R: GCTCCTTCTAAGCAAATCACTCC	

### Flow cytometry analysis

The equine macrophages were infected with pathogens as described above. The cells were digested with ACCUTASE cell digestion solution and centrifuged at 1,000 rpm for 10 min. After precipitation, the cells were fixed and blocked. The purified monoclonal antibody (1:100) was used as the primary antibody at 4°C overnight, and the FITC-conjugated goat anti-mouse IgG (1:200) was the secondary antibody. To establish baseline fluorescence and ensure rigorous gating, both unstained cells and secondary-antibody-only samples were included as negative controls in every experiment. Flow cytometry analysis was performed on an Accuri C6 flow cytometer (Becton-Dickinson). The gating strategy involved an initial morphological selection of the macrophage population using forward scatter (FSC) and side scatter (SSC) profiles to effectively exclude cellular debris. For quantitative reporting, the positive fluorescence threshold was strictly delineated based on the baseline established by the secondary-antibody-only control. Final quantifications are defined and reported as the percentage of specifically stained CD80-positive or CD163-positive cells within the primary FSC/SSC gated macrophage population.

### Western blotting

The equine macrophages were infected with pathogens as described above. The cells were washed with PBS, lysed in lysis buffer (50 mM HEPES-NaOH, pH 7.9, 100 mM NaCl, 50 mM KCl, 0.25% NP-40, 1 mM dithiothreitol), and centrifuged at 10,000 rpm for 5 min. Cell lysate was mixed with 5 × SDS protein loading buffer and boiled for 10 min at 95°C. Samples were separated using SDS-PAGE and transferred to polyvinyl difluoride (PVDF) membranes (Millipore). After blocking with 5% skim milk, the membranes were incubated with the purified monoclonal antibody (1:100) at 4°C overnight, and the DyLight-labeled goat anti-mouse IgG (1:5,000) was then applied as the secondary antibody for 1 h. After washing three times in TBST, the membranes were visualized using the Odyssey Infrared Imaging System (LI-COR, U.S.A.), and macrophages without infection were set as negative controls.

### Statistical analysis

All graphs were generated using GraphPad Prism version 6.0 (La Jolla, CA, USA). Primary equine macrophages were derived from peripheral blood mononuclear cells isolated from three healthy equine donors. Each experiment was performed with a minimum of three independent biological replicates (*n* = 3). For the quantitative analysis of western blot densitometry and flow cytometry cell percentages, statistical significance was evaluated using a two-tailed Student’s *t*-test (for comparing two groups, such as mock vs. infected) or a one-way analysis of variance, followed by a post-hoc test (for multiple comparisons across time points). All data are presented as the mean ± standard error of the mean (SEM). Asterisks denote statistical significance: NS, no significance; *, *P* < 0.05; **, *P* < 0.01; ***, *P* < 0.001.

## Data Availability

All data supporting the findings of this study are available within the article.

## SUPPLEMENTAL MATERIAL

Figure S1 spectrum.02916-25-s0001.tif Quantification of nitric oxide (NO) and inducible nitric oxide synthase (iNOS/Nos2) in macrophages and antibody production workflow.

Figure S2 spectrum.02916-25-s0002.tif Flow cytometric analysis of macrophage polarization.

Figure S3 spectrum.02916-25-s0003.tif Specificity validation of CD80 and CD163 antibodies.

Figure S4 spectrum.02916-25-s0004.tif Verification of the co-localization of CD14 and Macrophage Polarization Markers.

Supplemental Figure Legends spectrum.02916-25-s0005.docx Legends for Figures S1 to S4

ASM does not own the copyrights to Supplemental Material that may be linked to, or accessed through, an article. The authors have granted ASM a non-exclusive, world-wide license to publish the Supplemental Material files. Please contact the corresponding author directly for reuse.

## References

[1] Bain CC, Schridde A. 2018. Origin, differentiation, and function of intestinal macrophages. Front Immunol 9:2733. doi:10.3389/fimmu.2018.02733 30538701 PMC6277706

[2] Epelman S, Lavine KJ, Randolph GJ. 2014. Origin and functions of tissue macrophages. Immunity 41:21–35. doi:10.1016/j.immuni.2014.06.013 25035951 PMC4470379

[3] Cline TD, Beck D, Bianchini E. 2017. Influenza virus replication in macrophages: balancing protection and pathogenesis. J Gen Virol 98:2401–2412. doi:10.1099/jgv.0.000922 28884667 PMC5725990

[4] Locati M, Curtale G, Mantovani A. 2020. Diversity, mechanisms, and significance of macrophage plasticity. Annu Rev Pathol 15:123–147. doi:10.1146/annurev-pathmechdis-012418-012718 31530089 PMC7176483

[5] Sharifiaghdam M, Shaabani E, Faridi-Majidi R, De Smedt SC, Braeckmans K, Fraire JC. 2022. Macrophages as a therapeutic target to promote diabetic wound healing. Mol Ther 30:2891–2908. doi:10.1016/j.ymthe.2022.07.016 35918892 PMC9482022

[6] Orecchioni M, Ghosheh Y, Pramod AB, Ley K. 2019. Macrophage polarization: different gene signatures in M1(LPS+) vs. Classically and M2(LPS-) vs. Alternatively activated macrophages. Front Immunol 10:1084. doi:10.3389/fimmu.2019.01084 31178859 PMC6543837

[7] Cutolo M, Campitiello R, Gotelli E, Soldano S. 2022. The Role of M1/M2 macrophage polarization in rheumatoid arthritis synovitis. Front Immunol 13:867260. doi:10.3389/fimmu.2022.867260 35663975 PMC9161083

[8] O’Shea JJ, Murray PJ. 2008. Cytokine signaling modules in inflammatory responses. Immunity 28:477–487. doi:10.1016/j.immuni.2008.03.002 18400190 PMC2782488

[9] Mills CD, Kincaid K, Alt JM, Heilman MJ, Hill AM. 2000. M-1/M-2 macrophages and the Th1/Th2 paradigm. J Immunol 164:6166–6173. doi:10.4049/jimmunol.164.12.6166 10843666

[10] Menarim BC, Gillis KH, Oliver A, Ngo Y, Werre SR, Barrett SH, Rodgerson DH, Dahlgren LA. 2020. Macrophage activation in the synovium of healthy and osteoarthritic equine joints. Front Vet Sci 7:568756. doi:10.3389/fvets.2020.568756 33324696 PMC7726135

[11] Ka MB, Daumas A, Textoris J, Mege JL. 2014. Phenotypic diversity and emerging new tools to study macrophage activation in bacterial infectious diseases. Front Immunol 5:500. doi:10.3389/fimmu.2014.00500 25346736 PMC4193331

[12] Labonte AC, Tosello-Trampont AC, Hahn YS. 2014. The role of macrophage polarization in infectious and inflammatory diseases. Mol Cells 37:275–285. doi:10.14348/molcells.2014.2374 24625576 PMC4012075

[13] Tuo T, Chen D, Wang L, Zhang Y, Zhou L, Ge X, Han J, Guo X, Yang H. 2024. Infection of PRRSV inhibits CSFV C-strain replication by inducing macrophages polarization to M1. Vet Microbiol 289:109957. doi:10.1016/j.vetmic.2023.109957 38160508

[14] Saha B, Kodys K, Szabo G. 2016. Hepatitis C virus-induced monocyte differentiation into polarized M2 macrophages promotes stellate cell activation via TGF-β. Cell Mol Gastroenterol Hepatol 2:302–316. doi:10.1016/j.jcmgh.2015.12.005 28090562 PMC5042356

[15] Sun H, Sun Y, Pu J, Zhang Y, Zhu Q, Li J, Gu J, Chang KC, Liu J. 2014. Comparative virus replication and host innate responses in human cells infected with three prevalent clades (2.3.4, 2.3.2, and 7) of highly pathogenic avian influenza H5N1 viruses. J Virol 88:725–729. doi:10.1128/JVI.02510-13 24131718 PMC3911739

[16] Knoll R, Schultze JL, Schulte-Schrepping J. 2021. Monocytes and macrophages in COVID-19. Front Immunol 12:720109. doi:10.3389/fimmu.2021.720109 34367190 PMC8335157

[17] Cui L, Ma Y, Liang Y, Zhang Y, Chen Z, Wang Z, Wu H, Li X, Xu L, Liu S, Li H. 2021. Polarization of avian macrophages upon avian flavivirus infection. Vet Microbiol 256:109044. doi:10.1016/j.vetmic.2021.109044 33836389

[18] Zhang W, Fu Z, Yin H, Han Q, Fan W, Wang F, Shang Y. 2021. Macrophage polarization modulated by porcine circovirus type 2 facilitates bacterial coinfection. Front Immunol 12:688294. doi:10.3389/fimmu.2021.688294 34394082 PMC8355693

[19] Sebastian R, Sravanthi M, Umapathi V, Krishnaswamy N, Priyanka M, Dechamma HJ, Ganesh K, Basagoudanavar SH, Sanyal A, Reddy GR. 2020. Foot and mouth disease virus undergoes non-progressive replication in mice peritoneal macrophages and induces M1 polarization. Virus Res 281:197906. doi:10.1016/j.virusres.2020.197906 32109526 PMC7114663

[20] Jaslow SL, Gibbs KD, Fricke WF, Wang L, Pittman KJ, Mammel MK, Thaden JT, Fowler VG, Hammer GE, Elfenbein JR, Ko DC. 2018. *Salmonella* activation of STAT3 signaling by SarA effector promotes intracellular replication and production of IL-10. Cell Rep 23:3525–3536. doi:10.1016/j.celrep.2018.05.072 29924996 PMC6314477

[21] Karagianni AE, Kapetanovic R, Summers KM, McGorum BC, Hume DA, Pirie RS. 2017. Comparative transcriptome analysis of equine alveolar macrophages. Equine Vet J 49:375–382. doi:10.1111/evj.12584 27096353 PMC5412682

[22] Karagianni AE, Lisowski ZM, Hume DA, Scott Pirie R. 2021. The equine mononuclear phagocyte system: the relevance of the horse as a model for understanding human innate immunity. Equine Vet J 53:231–249. doi:10.1111/evj.13341 32881079

[23] Wilson ME, McCandless EE, Olszewski MA, Robinson NE. 2020. Alveolar macrophage phenotypes in severe equine asthma. Vet J 256:105436. doi:10.1016/j.tvjl.2020.105436 32113585 PMC7768773

[24] Dakin SG, Werling D, Hibbert A, Abayasekara DRE, Young NJ, Smith RKW, Dudhia J. 2012. Macrophage sub-populations and the lipoxin A4 receptor implicate active inflammation during equine tendon repair. PLoS One 7:e32333. doi:10.1371/journal.pone.0032333 22384219 PMC3284560

[25] Cao X, van den Hil FE, Mummery CL, Orlova VV. 2020. Generation and functional characterization of monocytes and macrophages derived from human induced pluripotent stem cells. CP Stem Cell Biology 52:e108. doi:10.1002/cpsc.108 PMC7154707 32159928

[26] Zhou Y, Yoshida S, Kubo Y, Yoshimura T, Kobayashi Y, Nakama T, Yamaguchi M, Ishikawa K, Oshima Y, Ishibashi T. 2017. Different distributions of M1 and M2 macrophages in a mouse model of laser-induced choroidal neovascularization. Mol Med Rep 15:3949–3956. doi:10.3892/mmr.2017.6491 28440413 PMC5436148

[27] Greenwald RJ, Freeman GJ, Sharpe AH. 2005. The B7 family revisited. Annu Rev Immunol 23:515–548. doi:10.1146/annurev.immunol.23.021704.115611 15771580

[28] Crespo H, Bertolotti L, Juganaru M, Glaria I, de Andrés D, Amorena B, Rosati S, Reina R. 2013. Small ruminant macrophage polarization may play a pivotal role on lentiviral infection. Vet Res 44:83. doi:10.1186/1297-9716-44-83 24070317 PMC3850683

[29] Trentin L, Zambello R, Sancetta R, Facco M, Cerutti A, Perin A, Siviero M, Basso U, Bortolin M, Adami F, Agostini C, Semenzato G. 1997. B lymphocytes from patients with chronic lymphoproliferative disorders are equipped with different costimulatory molecules. Cancer Res 57:4940–4947. 9354461

[30] Kim JK, Fahad AM, Shanmukhappa K, Kapil S. 2006. Defining the cellular target(s) of porcine reproductive and respiratory syndrome virus blocking monoclonal antibody 7G10. J Virol 80:689–696. doi:10.1128/JVI.80.2.689-696.2006 16378972 PMC1346842

[31] Law SK, Micklem KJ, Shaw JM, Zhang XP, Dong Y, Willis AC, Mason DY. 1993. A new macrophage differentiation antigen which is a member of the scavenger receptor superfamily. Eur J Immunol 23:2320–2325. doi:10.1002/eji.1830230940 8370408

[32] Oh D, Xie J, Vanderheijden N, Nauwynck HJ. 2020. Isolation and characterization of a new population of nasal surface macrophages and their susceptibility to PRRSV-1 subtype 1 (LV) and subtype 3 (Lena). Vet Res 51:21. doi:10.1186/s13567-020-00751-7 32093748 PMC7038536

[33] Sánchez C, Doménech N, Vázquez J, Alonso F, Ezquerra A, Domínguez J. 1999. The porcine 2A10 antigen is homologous to human CD163 and related to macrophage differentiation. J Immunol 162:5230–5237. 10227997

[34] Watson CJ, Glezeva N, Horgan S, Gallagher J, Phelan D, McDonald K, Tolan M, Baugh J, Collier P, Ledwidge M. 2020. Atrial tissue pro-fibrotic M2 macrophage marker CD163+, gene expression of procollagen and B-type natriuretic peptide. J Am Heart Assoc 9:e013416. doi:10.1161/JAHA.119.013416 32431194 PMC7428985

[35] Yamanishi S, Nagashima H, Tanaka K, Uno T, Ikeuchi Y, Iwahashi H, Hashiguchi M, Horii S, Itoh T, Muragaki Y, Sasayama T. 2025. Association of preoperative seizures with reduced expression of soluble CD163, an M2 macrophage marker, in the cerebrospinal fluid in isocitrate dehydrogenase wild-type glioblastoma. J Neurooncol 171:95–103. doi:10.1007/s11060-024-04837-6 39377994

[36] McWhorter FY, Wang T, Nguyen P, Chung T, Liu WF. 2013. Modulation of macrophage phenotype by cell shape. Proc Natl Acad Sci USA 110:17253–17258. doi:10.1073/pnas.1308887110 24101477 PMC3808615

[37] Hourani T, Perez-Gonzalez A, Khoshmanesh K, Luwor R, Achuthan AA, Baratchi S, O’Brien-Simpson NM, Al-Hourani A. 2023. Label-free macrophage phenotype classification using machine learning methods. Sci Rep 13:5202. doi:10.1038/s41598-023-32158-7 36997576 PMC10061362

[38] Pasqualini R, Arap W. 2004. Hybridoma-free generation of monoclonal antibodies. Proc Natl Acad Sci USA 101:257–259. doi:10.1073/pnas.0305834101 14688405 PMC314172

[39] Beyer M, Mallmann MR, Xue J, Staratschek-Jox A, Vorholt D, Krebs W, Sommer D, Sander J, Mertens C, Nino-Castro A, Schmidt SV, Schultze JL. 2012. High-resolution transcriptome of human macrophages. PLoS One 7:e45466. doi:10.1371/journal.pone.0045466 23029029 PMC3448669

[40] Tarique AA, Logan J, Thomas E, Holt PG, Sly PD, Fantino E. 2015. Phenotypic, functional, and plasticity features of classical and alternatively activated human macrophages. Am J Respir Cell Mol Biol 53:676–688. doi:10.1165/rcmb.2015-0012OC 25870903

[41] da Silva TB, Rendra E, David CAW, Bieback K, Cross MJ, Wilm B, Liptrott NJ, Murray P. 2023. Umbilical cord mesenchymal stromal cell-derived extracellular vesicles lack the potency to immunomodulate human monocyte-derived macrophages *in vitro*. Biomed Pharmacother 167:115624. doi:10.1016/j.biopha.2023.115624 37783151

[42] Piatnitskaia S, Rafikova G, Bilyalov A, Chugunov S, Akhatov I, Pavlov V, Kzhyshkowska J. 2024. Modelling of macrophage responses to biomaterials *in vitro*: state-of-the-art and the need for the improvement. Front Immunol 15:1349461. doi:10.3389/fimmu.2024.1349461 38596667 PMC11002093

[43] Costantini A, Viola N, Berretta A, Galeazzi R, Matacchione G, Sabbatinelli J, Storci G, De Matteis S, Butini L, Rippo MR, Procopio AD, Caraceni D, Antonicelli R, Olivieri F, Bonafè M. 2018. Age-related M1/M2 phenotype changes in circulating monocytes from healthy/unhealthy individuals. Aging (Albany NY) 10:1268–1280. doi:10.18632/aging.101465 29885276 PMC6046240

[44] Salei N, Rambichler S, Salvermoser J, Papaioannou NE, Schuchert R, Pakalniškytė D, Li N, Marschner JA, Lichtnekert J, Stremmel C, Cernilogar FM, Salvermoser M, Walzog B, Straub T, Schotta G, Anders H-J, Schulz C, Schraml BU. 2020. The kidney contains ontogenetically distinct dendritic cell and macrophage subtypes throughout development that differ in their inflammatory Properties. J Am Soc Nephrol 31:257–278. doi:10.1681/ASN.2019040419 31932472 PMC7003301

[45] Du C, Duan Y, Wang XF, Lin Y, Na L, Wang X, Chen K, Wang X. 2019. Attenuation of equine lentivirus alters mitochondrial protein expression profile from inflammation to apoptosis. J Virol 93:e00653-19. doi:10.1128/JVI.00653-19 31391270 PMC6803291

[46] Codo AC, Davanzo GG, Monteiro L de B, de Souza GF, Muraro SP, Virgilio-da-Silva JV, Prodonoff JS, Carregari VC, de Biagi Junior CAO, Crunfli F, et al.. 2020. Elevated glucose levels favor SARS-CoV-2 infection and monocyte response through a HIF-1α/glycolysis-dependent axis. Cell Metab 32:437–446. doi:10.1016/j.cmet.2020.07.007 32697943 PMC7367032

[47] Min CK, Shakya AK, Lee BJ, Streblow DN, Caposio P, Yurochko AD. 2020. The differentiation of human cytomegalovirus infected-monocytes is required for viral replication. Front Cell Infect Microbiol 10:368. doi:10.3389/fcimb.2020.00368 32850474 PMC7411144

[48] Viola A, Munari F, Sánchez-Rodríguez R, Scolaro T, Castegna A. 2019. The metabolic signature of macrophage responses. Front Immunol 10:1462. doi:10.3389/fimmu.2019.01462 31333642 PMC6618143

[49] Lim WS, Edwards JF, Boyd NK, Payne SL, Ball JM. 2003. Simultaneous quantitation of equine cytokine mRNAs using a multi-probe ribonuclease protection assay. Vet Immunol Immunopathol 91:45–51. doi:10.1016/s0165-2427(02)00263-5 12507849

[50] Muhsin A, Rangel R, Vien L, Bover L. 2022. Monoclonal antibodies generation: updates and protocols on hybridoma technology. Methods Mol Biol 2435:73–93. doi:10.1007/978-1-0716-2014-4_6 34993940

[51] Rao X, Huang X, Zhou Z, Lin X. 2013. An improvement of the 2ˆ(-delta delta CT) method for quantitative real-time polymerase chain reaction data analysis. Biostat Bioinforma Biomath 3:71–85. 25558171 PMC4280562

