# Supplementary material for: Systematic establishment of approaches to the detection of equine macrophage polarization and their application in pathogenic infection
Source: Microbiol Spectr. 2026 Jul 6;14(8):e02916-25. doi: 10.1128/spectrum.02916-25 (PMC13436364; doi:10.1128/spectrum.02916-25)
Supplement: Supplementary file 1 [file spectrum.02916-25-s0005.docx]

**Figure S1.** Quantification of nitric oxide (NO) and inducible nitric oxide synthase (iNOS/Nos2) in macrophages and antibody production workflow. (A) Equine macrophages were incubated for 24 hr in RPMI medium in the presence of IFN-γ/LPS or IL-10. Detection of intracellular nitric oxide (NO) in cells using DAF-FM. (B) Analysis of the Mean Fluorescence Intensity (MFI) of DAF-FM; (C) Quantification of Nos2 mRNA expression by qPCR. (D) Schematic illustration of the monoclonal antibody production process. (E) ELISA analysis of serum antibody titers post-CD80 immunization. (F) ELISA analysis of serum antibody titers post-CD163 immunization. (G) Whole-gel Western blot analysis of endogenous CD80 protein in LPS/IFN-γ-stimulated macrophages using an anti-CD80 monoclonal antibody. (H) Whole-gel Western blot analysis of endogenous CD163 protein in IL-10 stimulated macrophages using an anti-CD163 monoclonal antibody.

**Figure S2.** Flow cytometric analysis of macrophage polarization. (A) Flow cytometry analysis was performed to determine the proportion of CD80-positive cells after LPS/IFN-γ treatment for 12 h, 24 h, 36 h, and 48 h. (B) Flow cytometry analysis was performed to determine the proportion of CD163-positive cells after IL-10 treatment for 12 h, 24 h, 36 h, and 48 h.

**Figure S3.** Specificity validation of CD80 and CD163 antibodies. (A) Specificity validation of the CD80 antibody by Western blot analysis in human (HEK293, THP-1) and murine (RAW 264.7) cell lines. (B) Specificity validation of the CD163 antibody by Western blot analysis in human (HEK293, THP-1) and murine (RAW 264.7) cell lines. (C) qPCR validation of CD80 knockdown efficiency. (D) qPCR validation of CD163 knockdown efficiency. (E) Flow cytometry single-staining control with secondary antibody and validation after CD80 knockdown. (F) Flow cytometry single-staining control with secondary antibody and validation after CD163 knockdown.

**Figure S4.** Verification of the Co-localization of CD14 and Macrophage Polarization Markers. Equine macrophages were incubated for 24 h in RPMI medium containing either LPS/IFN-γ or IL-10 . (A) Cells were fixed and double-labeled with mouse anti-CD80 and rabbit anti-CD14 primary antibodies. Detection was performed using anti-mouse Alexa Fluor 488 (Green) for CD80 and anti-rabbit Alexa Fluor 555 (Red) for CD14. Nuclei were counterstained with DAPI (Blue). (B) Cells were fixed and double-labeled with mouse anti-CD163 and rabbit anti-CD14 primary antibodies. Detection was performed using anti-mouse Alexa Fluor 488 (Green) for CD163 and anti-rabbit Alexa Fluor 555 (Red) for CD14. Nuclei were counterstained with DAPI (Blue).
